# Differences in serum IgA responses to HIV-1 gp41 in elite controllers compared to viral suppressors on highly active antiretroviral therapy

**DOI:** 10.1371/journal.pone.0180245

**Published:** 2017-07-03

**Authors:** Rafiq Nabi, Zina Moldoveanu, Qing Wei, Elizabeth T. Golub, Helen G. Durkin, Ruth M. Greenblatt, Betsy C. Herold, Marek J. Nowicki, Seble Kassaye, Michael W. Cho, Abraham Pinter, Alan L. Landay, Jiri Mestecky, Pamela A. Kozlowski

**Affiliations:** 1 Department of Microbiology, Immunology and Parasitology, Louisiana State University Health Sciences Center, New Orleans, LA, United States of America; 2 Department of Microbiology, University of Alabama at Birmingham, Birmingham, AL, United States of America; 3 Department of Epidemiology, Johns Hopkins Bloomberg School of Public Health, Baltimore, MD, United States of America; 4 Departments of Pathology and Medicine, SUNY Downstate, Brooklyn, NY, United States of America; 5 Departments of Medicine and Epidemiology/Biostastistics, University of California, San Francisco, CA, United States of America; 6 Department of Obstetrics and Gynecology and Women's Health, Albert Einstein College of Medicine, Bronx, NY, United States of America; 7 Department of Pediatrics, University of Southern California, Los Angeles, CA, United States of America; 8 Department of Medicine, Georgetown University, Washington, D.C., United States of America; 9 Department of Biomedical Sciences, Iowa State University, Ames, IA, United States of America; 10 Public Health Research Institute, Rutgers New Jersey Medical School, Newark, NJ, United States of America; 11 Department of Immunity and Emerging Pathogens, Rush University Medical Center, Chicago, IL, United States of America; 12 Institute of immunology and Microbiology 1st Faculty of Medicine, Charles University, Prague, Czech Republic; Emory University School of Medicine, UNITED STATES

## Abstract

Mechanisms responsible for natural control of human immunodeficiency type 1 (HIV) replication in elite controllers (EC) remain incompletely defined. To determine if EC generate high quality HIV-specific IgA responses, we used Western blotting to compare the specificities and frequencies of IgA to HIV antigens in serum of gender-, age- and race-matched EC and aviremic controllers (HC) and viremic noncontrollers (HN) on highly active antiretroviral therapy (HAART). Concentrations and avidity of IgA to HIV antigens were measured using ELISA or multiplex assays. Measurements for IgG were performed in parallel. EC were found to have stronger p24- and V1V2-specific IgG responses than HN, but there were no IgG differences for EC and HC. In contrast, IgA in EC serum bound more frequently to gp160 and gag proteins than IgA in HC or HN. The avidity of anti-gp41 IgA was also greater in EC, and these subjects had stronger IgA responses to the gp41 heptad repeat region 1 (HR1), a reported target of anti-bacterial RNA polymerase antibodies that cross react with gp41. However, EC did not demonstrate greater IgA responses to *E*. *coli* RNA polymerase or to peptides containing the shared LRAI sequence, suggesting that most of their HR1-specific IgA antibodies were not induced by intestinal microbiota. In both EC and HAART recipients, the concentrations of HIV-specific IgG were greater than HIV-specific IgA, but their avidities were comparable, implying that they could compete for antigen. Exceptions were C1 peptides and V1V2 loops. IgG and IgA responses to these antigens were discordant, with IgG reacting to V1V2, and IgA reacting to C1, especially in EC. Interestingly, EC with IgG hypergammaglobulinemia had greater HIV-specific IgA and IgG responses than EC with normal total IgG levels. Heterogeneity in EC antibody responses may therefore be due to a more focused HIV-specific B cell response in some of these individuals. Overall, these data suggest that development of HIV-specific IgA responses and affinity maturation of anti-gp41 IgA antibodies occurs to a greater extent in EC than in subjects on HAART. Future studies will be required to determine if IgA antibodies in EC may contribute in control of viral replication.

## Introduction

The design of HIV vaccines and improved therapies for existing infections would benefit greatly from the identification of humoral and cellular immune responses that prevent or control human immunodeficiency virus type 1 (HIV) infection. Toward this goal, the innate and adaptive immune responses in blood of HIV-infected elite controllers (EC) have been intensively investigated in the last decade. Many EC maintain CD4 T cells in a normal range and naturally suppress HIV replication to levels that are undetectable using conventional PCR [1]. Numerous immunological differences have been noted between EC and HIV-infected progressors [1, 2]. However, most of these could be attributed to the severe immune system dysfunction in the progressors as a result of prolonged high viremia and CD4 T cell loss. Therefore, it may be more informative to search for immunological differences between EC and chronically-infected individuals who also have undetectable viremia and similar numbers of CD4 T cells as a result of HAART.

When compared to "HAART controllers" (HC), EC have been found to have greater frequencies of gag-specific polyfunctional CD4 T cells in blood and the rectal mucosa [3, 4]. Approximately 70% of EC express MHC Class I alleles associated with control of HIV infection [2], and the induction of higher quality gag-specific CD8 T cells in these EC may be primarily responsible for suppressing viral replication [5, 6]. However, many subjects who require HAART also express these protective alleles [2]. The frequency of gag-specific CD8 T cells in EC is also highly variable, even among those with protective alleles. Some EC have very low or even no detectable HIV-specific CD8 T cells [5, 7]. In addition, 30% of EC have no protective alleles at all. Therefore, other immune effectors likely contribute to control of HIV infection in EC.

It is well established that EC do not have higher titers of broadly neutralizing antibodies [7–10]. However, multiple Fc-mediated antibody activities may act together to limit viral replication in EC. Recently, it was reported that IgG from EC mediates more Fc-dependent functions, such as antibody-dependent cellular cytoxicity (ADCC) by natural killer (NK) cells and phagocytosis by monocytes and neutrophils [11]. Both HIV-specific serum IgA and monomeric IgA monoclonal antibodies (mAbs) to the HIV gp120 or gp41 envelope (Env) glycoproteins have also been shown capable of mediating numerous antiviral functions, including neutralization [12, 13], phagocytosis [14], ADCC by mononuclear cells [15] and antibody-dependent cellular viral inhibition (ADCVI) by neutrophils [16]. The ability to eliminate virus or kill virus-infected cells through Fc-mediated mechanisms may explain why levels or avidity of SIV-specific IgA antibodies in serum have been correlated with control of infection against neutralization-resistant SIV in some NHP vaccine studies [17–19]. Functions of IgA in EC have not yet been reported, and it is not even known if EC have higher avidity Env-specific IgA, which would be expected to better trigger FcαR-mediated functions. In most HIV-infected individuals, including those on HAART, HIV-specific IgA has been presumed to play a minimal role in HIV infection simply because so little is produced [12, 20, 21]. High levels of HIV-specific IgA have been observed during acute infection [8, 22], especially those directed against gp41, which have been reported to appear first due to stimulation of intestinal commensal-specific B cells that cross-react with gp41 [22, 23]. However, even during acute infection, HIV-specific IgG concentrations are 100-fold greater than IgA, and while IgG antibodies continue to rise, the IgA antibodies decline [22].

Why HIV infection induces so little HIV-specific IgA is still an open question. Infection or mucosal immunization with viruses, such as influenza, rotavirus and poliovirus, induce excellent IgA responses in secretions as well as in sera [24–27]. An IgA immune evasion mechanism has been hypothesized since the HIV nef protein has been found to inhibit class-switching to IgA [28]. On the other hand, a lack of class-switching should reduce the levels of total and specific IgA, yet IgA levels and responses to other microbial antigens in HIV-infected individuals remain unaltered or even enhanced [29–33]. Therefore, restricted IgA responses to HIV may be due specifically due to the loss of HIV-specific CD4 T helper cells, which have been reported to be preferential targets of HIV infection [34].

As EC better maintain HIV-specific CD4 T cells than HAART recipients, they may develop higher quality HIV-specific IgA responses, including broader IgA responses to HIV antigens and higher avidity IgA antibodies. No information regarding mucosal IgA responses is available for EC, and studies of HIV-specific IgA in serum of EC are limited [8, 35, 36] and somewhat conflicting. For example, in one study, levels of anti-gp120 serum IgA were not found to differ in EC and progressors [35]. However, others have reported that EC better maintain levels of gp120-specific serum IgA antibodies than acutely- or chronically-infected individuals not on HAART [36]. In the latter study, gp120-specific IgA antibodies in progressors were also shown to interfere with HIV-specific serum IgG-mediated ADCC. Importantly, this was not observed for EC who had higher titers of anti-gp120 serum IgA. This clearly indicates that in humans, as in vaccinated NHP, not all Env-specific serum IgA antibodies are detrimental for control of immunodeficiency virus infection, as suggested by the finding that HIV vaccine recipients with high titers of Env-specific IgA had an increased risk of HIV acquisition in the RV144 canarypox prime/gp120 boost vaccine trial [37]. In addition, in RV144, it was serum IgA antibodies to a particular site in the gp120 constant 1 domain (C1) that were associated with risk of infection and later found to interfere with IgG-mediated ADCC in RV144 vaccinee plasma [38]. EC may potentially produce less of these "detrimental" IgA antibodies, or possibly more counteracting beneficial IgG antibodies, such as those directed against the gp120 V1V2 loops, which were associated with protection in RV144 [37, 39, 40], but have not yet been examined in EC.

As a first step toward characterizing the IgA in EC, we compared the specificities, concentrations, magnitude of responses, and avidity of HIV-specific serum IgA antibodies in EC to those in aviremic HC who had similar numbers of CD4 T cells. We additionally included a group of HAART noncontrollers (HN) with low CD4 T cell counts to determine if HIV-specific IgA responses are related to numbers of these cells. Measurements for IgG, including antibodies to C1 and V1V2, were performed in parallel. Our results demonstrate that infected subjects with low CD4 T cell counts exhibit poorest HIV-specific IgA responses overall. In addition, EC do develop greater IgA responses to specific proteins and regions of HIV when compared to subjects who have similar numbers of CD4 T cells but require HAART to control infection.

## Materials and methods

### Study subjects

**Fig 1:**
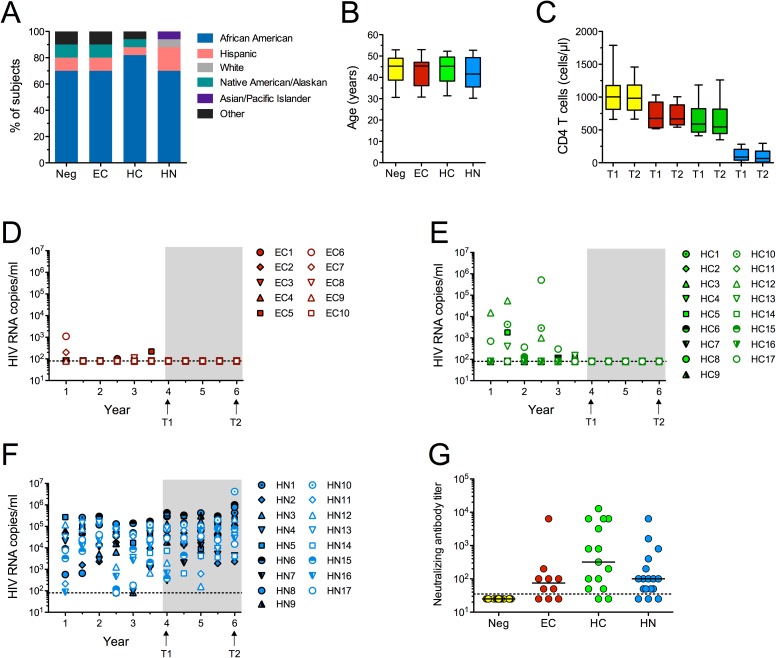
Characteristics of the study populations. Shown for each group of women enrolled in the study is the **(A)** racial composition, **(B)** age at the first time point (T1) of the study, and **(C)** CD4 T cell counts in blood at T1 and roughly 2 years later (T2). Whiskers in the box plots denote minimum to maximum values. The CD4 T cell counts in EC and HC did not differ significantly, and although they were lower than Neg controls (both p < 0.02), they were within the normal range (500-1500/μl). **(D-F)** Plasma viremia was measured by the WIHS at rough 6 month intervals and is shown for subjects starting 3 years before T1 and during the next 2 years. All VLs shown for HN and HC were measured while the subjects were on HAART. The dashed line represents the limit of detection in the PCR assay. **(G)** Neutralization of Clade C HIV_QZ4589_ (Trinidad) at T1 was measured using the TZM-bl assay. Data are expressed as the last reciprocal dilution of serum able to inhibit infection by 50%. Bars denote medians. Samples under the dashed line did not neutralize at the 1/50 starting dilution. No significant differences were found between any of the infection groups.

Two serum samples, collected at a 2 year interval (T1 and T2), were obtained from HIV uninfected women (Neg; n = 20) and from 3 groups of HIV-infected participants of the Women's Interagency HIV Study (WIHS), an observational cohort study of HIV infection in the United States. Plasma HIV RNA and blood CD4 T cell counts were measured by the WIHS as previously described [41]. The HIV-infected women were classified as EC (n = 10), HC (n = 17) or HAART noncontrollers (HN; n = 17) based on longitudinal viral loads (VL) and CD4 T cell counts. Women in all groups were matched as closely as possible for race and age. The majority were African-American, aged 40–48 years (Fig 1A and 1B). At T1, geometric mean CD4 T cell counts for Neg, EC, HC, and HN were 1005, 698, 626 and 81 per μl blood, respectively, and these numbers did not change significantly during the course of the study (Fig 1C). Women in the EC group had HIV RNA levels < 80 copies/ml, with not uncommon [42] occasional low viral blips (< 1500 RNA copies/ml) for 6 years prior to T1 and throughout the 2 year study in the absence of medications. For simplicity, VL in these and other infected women are only shown up to 3 years before T1 (Fig 1D). Women in the HC group controlled viral infection for a mimimum of 1 year before T1, but the majority (53%) were aviremic up to 3 years before the study (Fig 1E). All EC and HC remained aviremic throughout the 2 year study (Fig 1D and 1E). Women in the HN group were unable to consistently control viral replication for at least 3 years before T1 and were viremic during the 2 year study period (Fig 1F), most likely as a result of inconsistent adherence to drug regimens. None of the HC or HN had received early HAART (during or immediately after acute infection). Most did not begin combination therapy until their CD4 T cell counts had dropped below 350/μl (S1 Table). The date of HIV infection acquisition was not known for these subjects, but most had been infected for at least 10 years. Like other previously described EC cohorts [7–9], the EC in this study did not have higher titers of broadly neutralizing antibodies than HAART recipients (Fig 1G).

### Ethics statement

Informed consent for collection of blood was obtained from all study subjects. Approval for the study was received from the Louisana State University Health Sciences Center—New Orleans Institutional Review Board (Identification number 7819).

### Neutralization

Neutralizing assays were performed as described [43] using TZM-bl indicator cells and the Clade C QZ4589 Trinidad virus (both from the NIH AIDS Reagent Program: ARP). Briefly, diluted serum samples or medium alone were incubated in black, clear-bottom 96 well plates (Sigma, St Louis, MO) with 200 TCID_50_ of the Trinidad virus, which had been expanded in the CCR5+ C8166 T cell line (kindly provided by Dr. James Robinson, Tulane Medical School, New Orleans). After 30 min, 10^4^ TZM cells in medium containing DEAE dextran (Sigma) were added. Blank wells consisted of cells and medium alone. After 2 days at 5% CO_2_ and 37°C, the medium was removed and 75 μl of 0.1% Triton X-100 in PBS was added. After 10 min, 25 μl of Brite-Glo (Promega, Madison, WI) was added and relative light units (RLU) were recorded. The RLU in blank wells was subtracted. The last dilution of serum able to inhibit infection by 50% was then determined by comparing the RLU in test wells to that in wells given virus and cells alone.

### Western blotting

For Western blotting (WB), nitrocellulose strips containing electrophoretically separated HIV_IIIB_ antigens (Cambridge Biotech, Worcester, MA) were used to evaluate HIV-specific IgA and IgG antibodies in coded serum samples as previously described [44]. Briefly, strips were reacted overnight at 4°C with serum samples diluted 1/500 (for IgG) or 1/200 (for IgA). The following day, strips were washed and developed using biotinylated affinity-purified goat F(ab')_2_ IgG specific for human IgA or IgG (Genway Biotech, San Diego, CA), extravidin-alkaline phosphatase (Sigma) and nitro blue tetrazolium chloride enhanced 5-bromo-4-chloro-3-indolyl phosphate substrate (BCIP/NBT; BioRad, Hercules, CA).

### Recombinant proteins and peptides

All proteins used in the study were recombinant. The gp120consensus B protein used to measure binding antibodies in ELISA was produced as described [45]. Clade B nef protein, gp41ΔTMBAL and the gp120consensus B protein used in avidity assays were from Immune Technology (New York, NY). The p24 and gp41IIIB proteins were from Prospec (East Brunswick, NJ). The above proteins were expressed in 293T cells, except for nef and gp41IIIB, which were produced in *E*. *coli*. Murine leukemia virus gp70 scaffolded gp120 V1V2 proteins (gp70-V1V2) with sequences representative of Clade B (Case A2), Clade A (A244/CM244.c01) and Clade C (Consensus C) were produced in 293T cells as described [40, 46]. The gp41-54Q protein encompassing both the gp41 membrane proximal external region (MPER) and the C-terminal heptad repeat region 2 (HR2) was produced in E. coli and comprised the C-terminal 54 amino acids (630–683) of the M group consensus gp41 ectodomain. It contains a short T7_Tag_ leader sequence followed by the HR2, the MPER and a 6x His tag at the C-terminus with a K to Q mutation just before the 6xHis tag. For measurement of C1 antibodies by ELISA the following gp120consensus B peptides (from ARP) were pooled: EQMHEDIISLWDQSL, EDIISLWDQSLKPCV, SLWDQSLKPCVKLTP, SLWDQSLKPCVKLTP, QSLKPCVKLTPPLCVT. The C1 peptide KKKMHEDIISLWDESLKPCVKLTPLCV used in binding antibody mutliplex assays (BAMA) was synthesized by NeoScientific (Cambridge, MA). The gp41 N-terminal heptad repeat region 1 (HR1) peptide IKKEIAIKKEQEAIKKIEAIEKEISGIVQQQNNLLRAIEAQQHLLQLTVWGIKQLQARVL (amino acids 546–581; underlined) was synthesized by Neoscientific with a coiled-coil extension at the N-terminus (non-underlined portion) to improve coiled-coil stability [47] and structural homology to the *E*. *coli* RNA polymerase alpha subunit, one identified target of gp41/microbiota cross-reactive antibodies [48]. *E*. *coli* RNA polymerase core enzyme was from NEN (Wellesley, MA). Overlapping 15-mer peptides spanning the HR1 region were from the ARP. The KQLQARVLAVERYLK (QLAVERY) peptide and the cysteine-bonded gp41 immunodominant loop domain (ID) peptide KKKDQQLLGMWGCSGKLIC (gp41 amino acids 589–604) were produced by Genscript (Piscataway, NJ). These regions of gp120 and the gp41 ectodomain have been illustrated and described by others [40, 49, 50].

### ELISA and BAMA for HIV-specific binding antibodies

Binding antibodies to gp120_consensus_ B were measured by ELISA as described [21] using plates coated with 100 ng of gp120 per well, with the exception of 2 rows which were coated with 100 ng per well of goat F(ab')_2_ IgG to human IgA or IgG (for capture of serum IgA or IgG reference standards). Serial dilutions of a normal human serum containing known amounts of total IgG and IgA and test sera were added to blocked plates and allowed to react overnight at 4°C. The following day, plates were developed with biotin-labeled goat F(ab')_2_ IgG to human IgG or IgA (Biosource, Camarillo, CA), Extravidin-conjugated peroxidase, o-phenylenediamine-H_2_O_2_ substrate (both Sigma) and 1M H_2_SO_4_ stop solution. Absorbance was recorded at 490 nm.

Binding antibodies to all other HIV proteins and peptides were quantitated by ELISA as described [51] or by BAMA. Prior to performing IgA assays, sera were depleted of IgG using Protein G Sepharose (GE Healthcare, Chicago, IL) as described [51]. For ELISA, plates were coated overnight with 100 ng per well of protein in PBS or 500 ng per well of peptide(s) in 0.1 M bicarbonate buffer, pH 9.6. Blocked plates were loaded with dilutions of standard and test serum, which were allowed to react overnight at 4°C. The standard in most IgA assays was an IgG-depleted HIV-positive pooled serum. For C1 and V1V2 IgA assays, the standard was a purified monomeric IgA myeloma protein directly coated onto plates. Pooled HIV-positive purified IgG was used as a standard in most IgG assays. Other standards and internal controls included a C1 peptide-specific rhesus macaque antiserum (generously provided by Dr. Georgia Tomaras, Duke Medical School), the ELDKWA-specific 2F5 IgG mAb (a gift from Dr. Herman Katinger, Polymun Scientific GmbH, Austria) and the gp41 ID-specific 7B2 IgG mAb (kindly provided by Dr. James E. Robinson). All standards were assigned concentrations based on their endpoint titers, with an endpoint titer of 1/4,000 corresponding to 1 μg/ml. This produced results analogous to those obtained by coating portions of plates with anti-human IgA or IgG as in the above gp120 ELISAs. Plates were developed using 0.1 μg/ml biotinylated anti-human IgA or IgG, 1/4000 Neutralite avidin-peroxidase, tetramethylbenzidine (TMB) substrate solution (all from Southern Biotech) and 1N H_2_SO_4_ stop solution. Absorbance was recorded at 450nm.

For BAMA, 200μg of C1, ID or HR1 peptide was conjugated to 10^7^ Bio-Plex magnetic carboxylated beads (BioRad) as decribed [52]. White 96 well microtiter plates were blocked for 2 h at room temperature with PBS containing 1% BSA, 0.05% Tween-20 and 0.05% azide (bead buffer). Block buffer was removed and 25 μl diluted serum or standard and 25 μl combined beads were added to each well. The plate was mixed overnight at 4°C and 600 rpm on an orbital shaker. The following day, the plates were alternatively washed with PBS containing 0.05% Tween-20 in a Bio-Plex Pro Wash Station (BioRad) and mixed for 30 min at 1100rpm on an Eppendorf Mixmate (Fisher Scientific) with bead buffer containing 2 μg/ml biotinylated goat anti-human IgA or IgG antibody, followed by 1/200 phycoerythrin-labeled Neutralite avidin (all Southern Biotech). After the final wash, beads were suspended in 100 μl of bead buffer and analyzed in a Bioplex 200 using Bioplex Manager software (BioRad).

### ELISA for total immunoglobulin levels

Concentrations of total IgA or IgG were measured as described [51] using plates coated with 0.25 μg/ml affinity-purified goat anti-human IgA or IgG (MP Biomedicals, Solon, OH). Standards were purified human IgG or serum (for IgA) that had been calibrated using the Human Ig calibrator (Binding Site, Birmingham, UK). Plates were developed using biotinylated affinity-purified goat anti-human IgA or -IgG antibodies (Southern Biotech), neutralite avidin-peroxidase and TMB substrate as described above.

### Antibody avidity

A sodium thiocyanate (NaSCN) displacement ELISA similar to that previously described [53] was used to measure the avidity of antibodies to gp120, gp70-V1V2, gp41, p24, HR1 peptide or ID peptide. Briefly, microtiter plates were coated overnight with antigen, washed and blocked as described above. Wells on each side of the plate were then loaded with 100 μl of identical dilutions of standard and test samples. Following an overnight reaction at 4°C, the plate was warmed to 37°C in an oven for 15 min. A 100 μl volume of freshly-prepared 3M NaSCN in PBS at 37°C was then added to one side of the plate. After 10 min at 37°C, the plate was washed and developed with TMB as described above. The avidity index (AI) was calculated by dividing the antibody concentration in NaSCN-treated wells by that in untreated wells, then multiplying by 100%. Samples with insignificant or very low concentrations of HIV-specific antibody (absorbance values < 0.5 at a 1/50 dilution) were excluded from these analyses. To confirm that the NaSCN treatment did not result in loss of the coating antigen from the plate, half of the coated/blocked plates were treated with NaSCN before standards and internal controls were added. After development, antibody concentrations in NaSCN-treated wells were found to be 98–100% of those measured in untreated wells, indicating that antigens had been retained (data not shown).

### Statistics

Statistical analyses were performed using GraphPad Prism 5 (GraphPad Software, La Jolla, CA) with the confidence interval set to 95%. Two-tailed Fisher's Exact test was used to compare the frequency of responses between groups. Within group comparisons were done using the nonparametric two-tailed Wilcoxon matched pairs test. Correlations were performed using two-tailed Spearman rank test. The Kruskal-Wallis test was used to determine if there were differences between groups. If a significant p value was obtained in the Kruskal-Wallis test, then the groups were subjected to pairwise comparisons using the two-tailed Mann-Whitney rank sum test. This approach was taken because Dunn's comparisons between 3 or more groups is not ideal when group sizes are small [54]. For within group comparisons of IgA antibody responses to individual HR1 peptides, the repeated measures nonparametric Friedman test followed by Dunn's post-hoc analysis was used. All tests were performed at the 95% confidence interval. Results were considered significant if p values ≤ 0.05 were obtained.

## Results

### Frequency of HIV-specific IgA antibodies in EC

**Fig 2:**
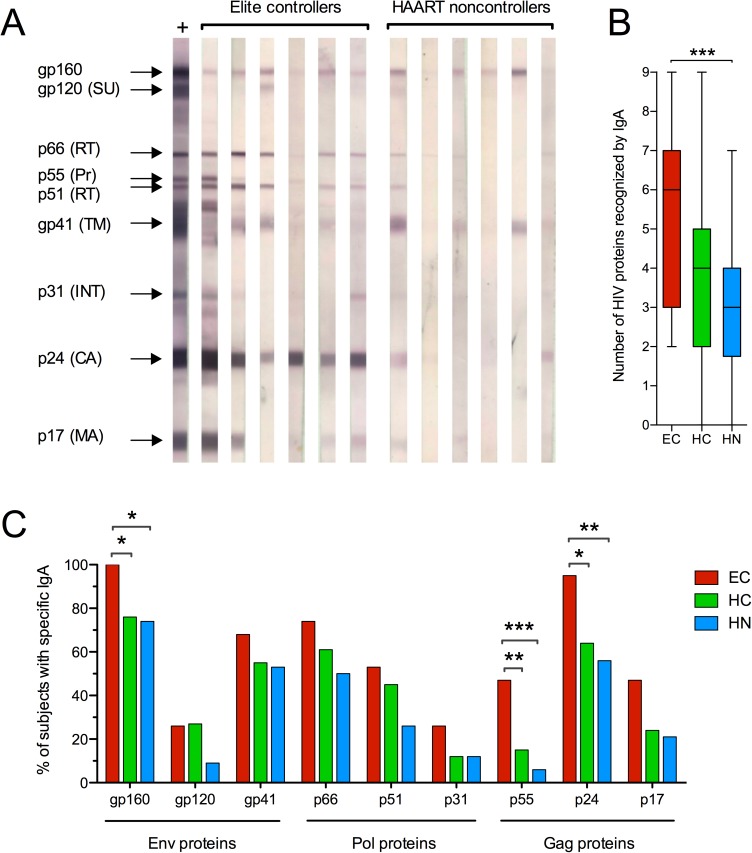
HIV proteins recognized by IgA. Western blotting was used to evaluate T1 and T2 serum IgA antibodies to HIV_IIIB_ Env antigens (gp160, gp120 surface unit and gp41 transmembrane protein), Pol proteins (p66 and p51 reverse transcriptase subunits, p31 integrase) and Gag proteins (p55 precursor, p24 capsid and p17 matrix). **(A)** Representative reactivity to HIV proteins by IgA in 6 different EC and HN subjects. The 9 HIV proteins on the strips are designated on the left using an HIV IgG positive control serum. **(B)** The number of HIV antigens bound by IgA at both T1 and T2 was compared using the Mann-Whitney test and is depicted in a Tukey box plot. **(C)** The percentage of subjects positive for IgA antibodies to each HIV protein at T1 and T2 was compared using Fisher's exact test. *p < 0.05; **p < 0.01; ***p < 0.001.

To determine if EC produce IgA antibodies to more HIV antigens than HN or HC, we used WB to evaluate antibody specificities in blinded T1 and T2 serum samples (Fig 2). After unblinding, it was visually apparent that IgA in EC bound more HIVIIIB proteins than IgA in HN (Fig 2A). When the total number of proteins recognized by IgA was compared, EC IgA was found to react with significantly more HIV antigens than HN IgA (Fig 2B). There was also a trend toward increased recognition of HIV proteins by EC IgA compared to HC IgA (p = 0.0554). When the IgA responses to individual proteins were evaluated, a significantly greater percentage of EC were found to have IgA that reacted with the gp160 Env glycoprotein and both p55 and p24 gag proteins when compared to HC as well as HN (Fig 2C). The IgG in EC also reacted more frequently with p55 than did IgG in HN (S1 Fig). However, this was the only difference we found for IgG antibodies in EC and HAART recipients using WB.

Overall, these results suggest that EC develop IgA responses to more HIV antigens than subjects on HAART, including those with similar numbers of CD4 T cells and undetectable viremia.

### Persistence of total and HIV-specific antibodies

**Fig 3:**
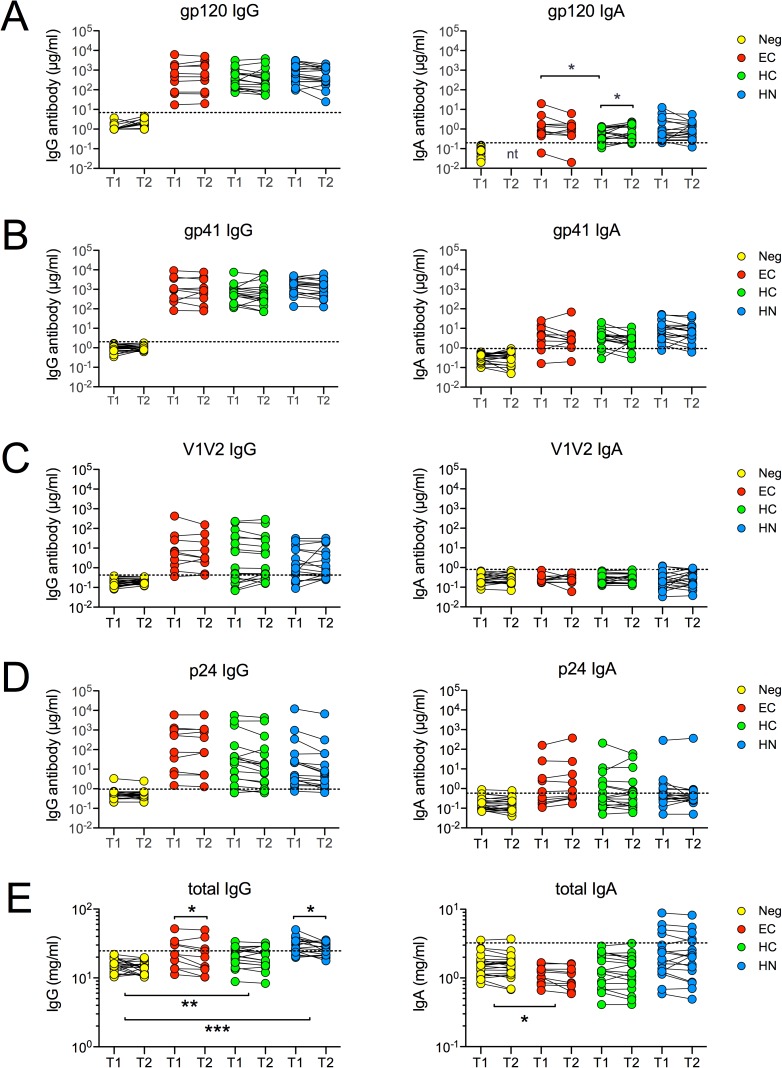
Persistence of IgA and IgG antibody concentrations. Concentrations of serum IgG and IgA antibodies to **(A)** gp120_consensus_ B, **(B)** gp41_BAL_ **(C)** gp70-V1V2_Clade B/Case A2_ and **(D)** p24_HXB2_ were quantitated at T1 and T2 using ELISA. **(E)** Total IgG and IgA concentrations at T1 and T2 were also measured by ELISA. Dashed lines denote the cut-offs for significance, which represent the mean + 3 SD of Neg control values at both time points, or at T1 alone if T2 was not tested (nt). In every infection group, concentrations of antigen-specific IgG or IgA were significantly greater than those in Neg controls (all p < 0.05) except for V1V2-specific IgA. At T1 and T2, concentrations of total IgG in HC or HN were also greater than those in Neg. At both T1 and T2, the total IgA in EC was lower than in Neg controls. Within group comparisons at T1 and T2 were done using the Wilcoxon matched pairs test. Between group comparisons at matched time points were performed using Mann-Whitney rank sum test. *p < 0.05; **p < 0.01; ***p < 0.001.

We next used ELISA to determine concentrations of antibodies to p24 and HIV Env antigens representative of CCR5-tropic Clade B viruses in sera collected at T1 and T2 (Fig 3). A gp70-V1V2 scaffolded protein was included in these analyses because high titers of serum IgG antibodies to gp70-V1V2 were associated with protection in the RV144 HIV vaccine trial [37].

All infection groups were found to have significantly greater concentrations of IgG to gp120, gp70-V1V2, gp41 and p24 as compared to Neg controls (all p < 0.0001). These IgG antibody concentrations did not change significantly from T1 to T2 within any of the groups (Fig 3A–3D). Concentrations of gp70-V1V2 IgG in EC tended to be higher than those in HN, but there were no significant differences between groups for concentrations of IgG antibodies to gp70-V1V2 or the other antigens tested.

Concentrations of HIV-specific IgA were also well-preserved within each group (Fig 3). The only difference found between T1 and T2 was a very modest 1.5-fold mean increase of anti-gp120 IgA in HC (Fig 3A). In EC, concentrations of anti-gp120 IgA were slightly higher than those in HC at T1 (p = 0.0205), but not at T2, and no other differences in HIV-specific IgA concentrations were observed between the groups at matched time points. When compared to Neg controls, all infection groups had significantly greater levels of IgA specific for each of the antigens tested, with the exception of gp70-V1V2. Only 1 infected subject had significant concentrations of gp70-V1V2-specific IgA, and this was extremely low (Fig 3C).

Total IgG and IgA concentrations were also measured (Fig 3E). Total IgA did not change from T1 to T2, but IgG did decline slightly in EC and HN (by 14% and 10%, respectively). Many HN were found to have IgG hypergammaglobulinemia (59%), and 24% of them also had elevated IgA. Many of the EC (40%) also had elevated IgG, but the total IgA in EC was slightly lower than Neg controls (Fig 3; p < 0.03 at T1 or T2). It should be noted, though, that total IgA concentrations in EC were not significantly lower than those in the HAART groups.

These results indicate that, despite longterm suppression of viral replication, EC develop and maintain concentrations of HIV-specific serum IgG and IgA that are comparable to those in both aviremic and viremic HAART subjects.

### Magnitude of HIV-specific antibody responses

**Fig 4:**
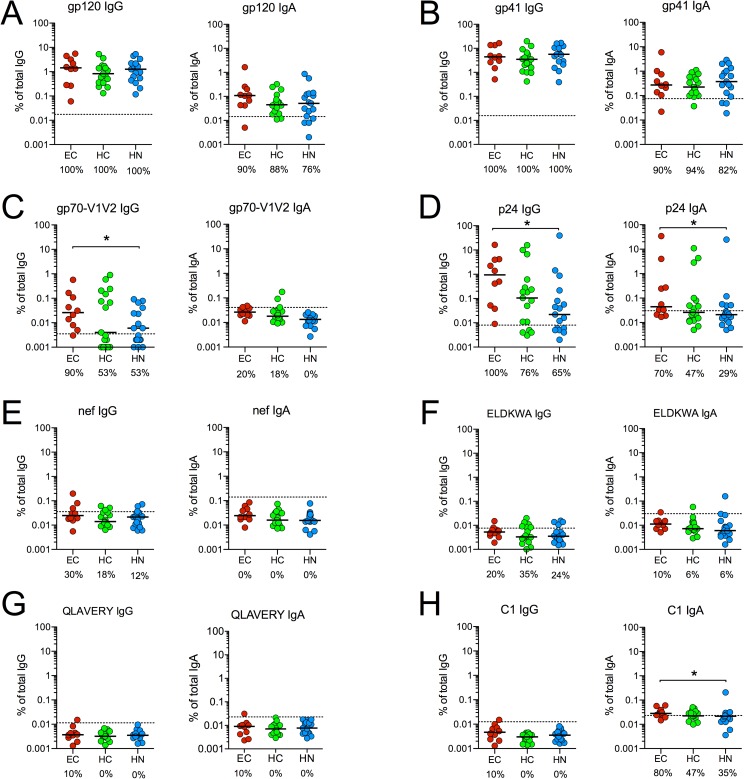
Magnitude of HIV-specific antibody responses. The average IgG and IgA sp. act. at T1 and T2 to **(A)** gp120 _consensus B_, **(B)** gp41_BAL_, **(C)** gp70-V1V2 _Case A2_, **(D)** p24_HXB2_, **(E)** nef and **(F)** gp41 ELDKWA peptide is presented as the percentage of antibody within each Ig isotype. Also shown is the sp. act. to **(G)** gp41 QLAVERY peptide at T1 and **(H)** C1 peptides at T2. Bars denote medians. Dashed lines represent the mean + 3SD of Neg controls. The frequency of subjects with positive responses is presented below each graph. *p < 0.05 by two-tailed Mann-Whitney rank sum test.

Next, we compared the magnitude of the HIV-specific IgA and IgG responses by expressing the HIV-specific antibodies as the percentage of the corresponding isotype. Namely, the concentrations (in μg/ml) of antigen-specific IgA or IgG were divided by the concentration (in μg/ml) of total IgA or IgG measured in each serum, then multiplied by 100. We termed these values specific activity (sp. act.). Because there were no, or only very modest differences between T1 and T2 concentrations of HIV-specific and total antibody levels within each group (Fig 3), no significant differences were found between the T1 and T2 sp. act. within the groups (not shown). Therefore, the average sp. act. for gp120, gp41, gp70-V1V2 and p24 is presented in Fig 4A–4D. The sp. act. to pooled C1 peptides, nef protein, and two gp41 peptides (ELDKWA and QLAVERY) at T1 and/or T2 was also determined. Antibody responses to the ELDKWA sequence in the gp41 membrane proximal external region (MPER) and the QLAVERY determinant in the gp41 HR1 were evaluated because these sequences have been reported to be recognized by the IgA in HIV-resistant individuals [55]. However, only 1/10 EC had IgA (or IgG) that reacted with these peptides (Fig 4F and 4G). Anti-nef antibodies, which can mediate ADCC [56] and have been associated with delayed disease progression [57], were also infrequently detected in EC (Fig 4E).

When the IgA sp. act. and frequency of IgA responses to other HIV antigens were compared between groups, the only differences found were that the IgA sp. act. to pooled C1 peptides and p24 in EC were higher than in HN (Fig 4D and 4H). This result for C1 was unexpected because results of the RV144 vaccine trial suggested that anti-C1 serum IgA antibodies may be detrimental and elevate the risk for HIV infection [37]. However, the greater anti-C1 IgA response in EC was also observed in BAMA (S2 Fig) with a single C1 peptide analogous to that tested in RV144 [37].

For IgG, the only differences found between EC and HAART recipients were that EC had higher IgG sp. act. to gp70-V1V2 and p24 when compared to HN (Fig 4C and 4D). Interestingly, subjects with the strongest IgG responses to Clade B gp70-V1V2 also reacted better to gp70-V1V2 proteins representative of other clades (S3 Fig), a finding similar to that observed in RV144 [40]. In contrast, IgA responses to gp70-V1V2 were rarely observed. Indeed, of the 44 infected subjects in this study, only 4 (2 EC and 2 HC) had significant IgA sp. act. to Clade B gp70-V1V2 (Fig 4C), and none had IgA responses to Clade A or Clade C V1V2 proteins (not shown).

In EC as well as HAART subjects, the IgG antibody responses to gp120, gp41 and p24 were dominant. For example, the magnitude of the IgG response to gp120 and gp41 was approximately 40- and 100-fold greater than the magnitude of the respective IgA responses to these proteins (S4 Fig). Despite these differences in the magnitude of IgG and IgA responses, the IgG and IgA sp. act. to gp120, gp41 and p24 were highly correlated (S5 Fig).

These data indicate that IgG dominates the humoral response to HIV in EC as well as in HAART recipients. They also show that, in EC, the magnitude of IgA antibody responses to HIV antigens, especially gag, can be quite heterogeneous.

### Antibody responses in subjects with IgG hypergammaglobulinemia

**Fig 5:**
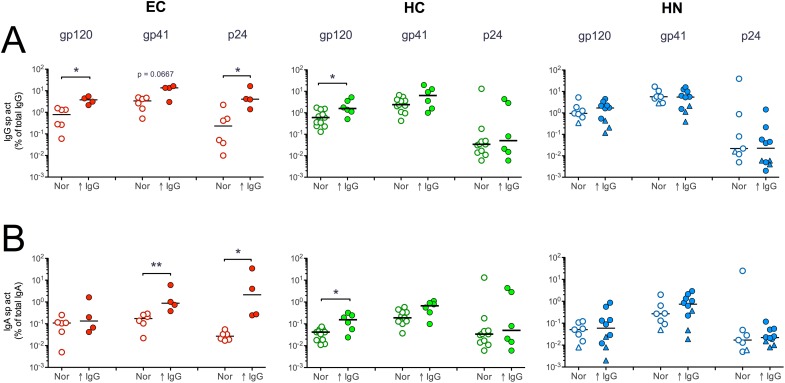
HIV-specific IgG and IgA responses in individuals with normal or elevated total IgG concentration. The gp120, gp41 and p24 sp. act. of **(A)** IgG and **(B)** IgA antibodies measured in subjects with normal levels of total IgG (Nor; open circles) is shown in comparison to those with IgG hypergammaglobulinemia (↑ IgG; closed circles). The triangles in the HN graphs represent the 4 subjects with IgA hypergammaglobulinemia. Bars are medians. *p < 0.05 or **p < 0.01 by Mann-Whitney rank sum test.

EC with high total serum IgG1 levels were recently reported to display greater levels of Env-specific IgG1 and Fc-mediated antiviral activity than EC with lower levels of total IgG1 [58]. Similarly, we observed that EC with IgG hypergammaglobulinemia typically had higher magnitude IgG responses to HIV proteins than EC with normal levels of total IgG (Fig 5A). Interestingly, EC with elevated IgG also had significantly geater IgA responses to gp41 and p24 than EC with normal levels of total IgG (Fig 5B). This was less evident in HC (Fig 5), and in HN with normal or elevated IgG; there were no differences in the IgG and IgA responses to Env and gag proteins (Fig 5). An exception were the HN with elevated total IgA. These subjects typically demonstrated the weakest IgG and IgA responses to HIV proteins (Fig 5), a finding consistent with the previous observation that IgA hypergammaglobulinemia is indicative of nonspecific polyclonal B cell activation [20].

Although the number of EC in our study is small, the differences between EC and HN with normal and elevated IgG were quite striking. Thus, in contrast to HAART subjects, IgG hypergammaglobulinemia in EC may reflect a highly specific antibody response to HIV, rather than nonspecific polyclonal activation.

### Avidity of HIV-specific IgA and IgG antibodies

**Fig 6:**
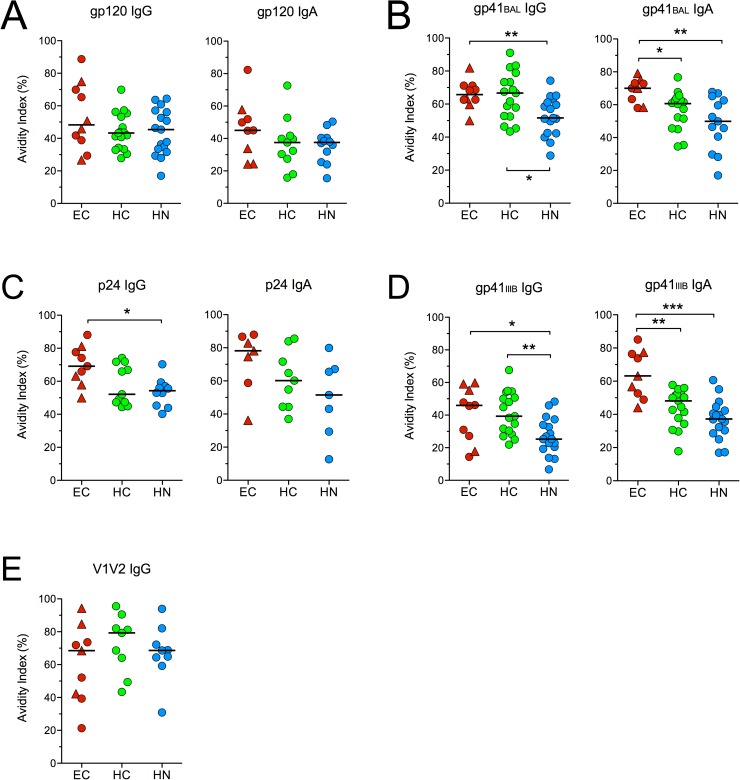
Avidity of HIV-specific IgG and IgA antibodies. The average avidity of serum IgG and IgA antibodies to **(A)** gp120_consensus B_, **(B)** 293T-derived gp41_BAL_, **(C)** p24, **(D)** *E*. *coli*-derived gp41_IIIB_ and **(E)** gp70-V1V2_Case A2_ at T1 and T2 is shown for subjects that had levels of binding antibodies that produced absorbance values > 0.5 at a 1/50 (for IgA) or 1/100 (for IgG) dilution. Bars represent medians. *p < 0.05, **p < 0.01 or ***p < 0.001 by two-tailed Mann-Whitney rank sum test.

To determine if EC have higher quality HIV-specific IgA antibodies than HAART recipients, we used the NaSCN displacement ELISA to measure the avidity of gp120, gp41, gp70-V1V2 and p24 antibodies in serum of subjects who had adequate levels of antibodies for these measurements. The avidity indices measured within each group did not change significantly from T1 to T2 (S6 Fig). Therefore, the average avidity at T1 and T2 was calculated and is shown in Fig 6.

Despite having higher magnitude IgG responses to HIV antigens, the avidities of IgA and IgG antibodies were similar within each group (Fig 6), though they infrequently correlated (S7 Fig). When IgG antibody avidities in EC were compared to HAART recipients, the EC were found to have higher avidity gp41- and p24-specific IgG than the HN, but no differences were observed between EC and HC for IgG antibody avidities (Fig 6B and 6C). Similarly, EC had higher avidity gp41-specific IgA and a trend toward higher avidity p24-specific IgA (p = 0.0728) when compared to HN (Fig 6B and 6C). However, the EC also had higher avidity gp41-specific IgA than the HC (Fig 6B). To confirm this, the avidity of antibodies to another gp41 protein, gp41IIIB, were measured (Fig 6D). Again, the avidity of anti-gp41 IgA (but not IgG) in EC was found to be greater than that in HC and HN. Since the gp41IIIB protein was produced in *E*. *coli*, this finding indicates that the higher avidity gp41-specific IgA antibodies in EC are not directed against N-glycans on the gp41 ectodomain.

These results demonstrate that Env-specific IgA antibodies produced by HIV-infected individuals could potentially compete with IgG for binding to HIV Env antigens despite their lower concentrations. The data additionally suggest that affinity maturation of gp41-specific IgA may occur to a greater extent in EC than in subjects on HAART.

### IgA responses to the gp41 ID and HR1 domain

**Fig 7:**
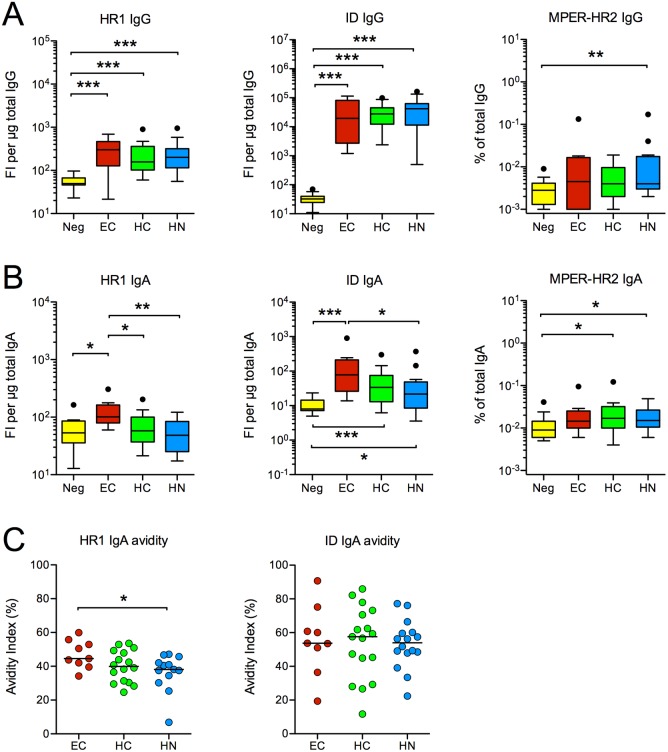
Specificity and avidity of gp41 antibodies. Tukey box plots illustrating the **(A)** IgG or **(B)** IgA sp. act. to gp41 HR1 full length peptide, gp41 ID cyclic peptide and the 54Q protein containing the MPER and HR2 regions at T2. Antibodies to 54Q were measured using ELISA; those to HR1 and ID were measured simultaneously using BAMA. The fluorescent intensity (FI) values obtained in assays for HR1- and ID-specific IgA or IgG were adjusted relative to the amount of total IgA or IgG in the diluted serum samples to obtain the sp. act. Note that the IgG responses in EC and subjects on HAART were not significantly different. **(C)** The avidity of anti-HR1 and anti-ID IgA antibodies was measured using the NaSCN displacement ELISA. *p < 0.05, **p < 0.01, ***p < 0.001 by two-tailed Mann-Whitney rank sum test.

In an effort to narrow down the determinant(s) that might be recognized by the higher-avidity anti-gp41 IgA in EC, we searched for regions of gp41 that were targeted by EC IgA to a greater extent than HC and HN IgA. From our earlier analyses (Fig 3), we knew that these determinants were not ELDKWA or QARLAVERY, as responses to these peptides were generally absent. We also observed infrequent IgA reactivity to a truncated gp41 protein that encompassed both the gp41 MPER and HR2 regions (Fig 7B). The ID region appeared to be a major target of the gp41-specific IgA in EC, and the IgA sp. act. to ID in EC was greater than that in HN (Fig 7B). However, the magnitude of IgA responses to the gp41 ID did not differ between EC and HC (Fig 7B). The avidity of ID-specific IgA also did not differ among the groups (Fig 7C). On the other hand, the magnitude of IgA responses to a peptide encompassing the entire HR1 domain was greater in EC when compared to either HC or HN (Fig 7B). The avidity of HR1-specific IgA was also higher in EC than HN (Fig 7C). These results suggest that the higher avidity gp41-specific IgA in EC compared to HN may be due to higher avidity HR1-specific IgA in the EC.

### IgA responses to bacterial RNA polymerase and HR1 peptides

**Fig 8:**
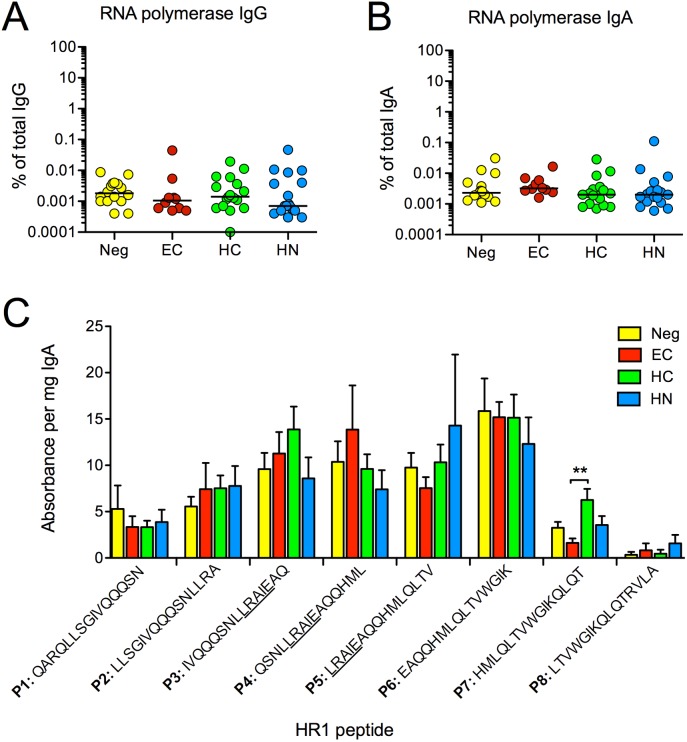
Antibody responses to bacterial RNA polymerase and HR1 peptides. **(A)** IgG and **(B)** IgA antibodies to *E*. *coli* RNA polymerase were measured by ELISA in T2 serum and are expressed as the % of antibodies within the total IgG or IgA. There were no significant differences between the groups. **(C)** IgA antibodies to HR1 overlapping peptides were measured by ELISA using a 1/50 serum dilution. Results for the QARLAVERY peptide are not presented since responses to this peptide were already shown to be absent. To determine the magnitude of peptide-specific IgA antibody responses, the absorbance measured for QARLAVERY peptide (the background control) was subtracted from the absorbance obtained for each peptide (P). The adjusted absorbance value was then divided by the concentration of total IgA in the diluted serum. No significant differences were found among the groups for responses to P1-7. HC did have greater IgA responses to P8 when compared to EC (**p < 0.01 by Mann-Whitney). Within group differences in IgA peptide reactivity are presented in S2 Table.

The HR1 peptide used in the above analysis encompasses the entire HR1 region and has been reported to form a coiled-coil conformation in solution [47]. Therefore, it should share not only the LRAI linear sequence but also conformational homology in the alpha subunit of *E*. *coli* RNA polymerase, reported targets of gp41/intestinal microbiota cross-reactive antibodies [48]. This raised the possibility that the stronger IgA responses to HR1 in EC might be related to induction of these cross-reactive antibodies. We therefore evaluated antibody responses to *E*. *coli* RNA polymerase and overlapping 15-mer HR1 peptides, 3 of which contained the LRAI sequence (Fig 8). IgA responses to RNA polymerase were extremely low in all subjects and did not differ among the groups, including uninfected women (Fig 8A and 8B). EC also failed to demonstrate greater IgA responses to any of the 8 HR1 peptides tested (Fig 8C). Unexpectedly, the IgA response to peptide 6 (P6), which lacked the LRAI sequence, dominated in all study populations (Fig 8C and S2 Table). These data indicate that the HR1-specific IgA antibodies in EC and other study subjects are not previously described gp41/intestinal microbiota cross-reactive antibodies. In addition, these results suggest that the higher magnitude and higher avidity IgA response measured against the whole HR1 domain in EC may be represented by conformational antibodies.

## Discussion

Comparative analyses of immune responses in EC to those in infected aviremic subjects who can only control HIV infection with HAART may provide clues as to the types of immune responses that should be generated by HIV vaccines to achieve optimal control of infection. In this study, we found that EC had HIV-specific serum IgA responses that differed from those in HC in several respects. The IgA in EC recognized more HIV proteins, especially gp160 Env and p55 and p24 Gag proteins. The EC also had higher-avidity IgA antibodies to gp41 and higher magnitude IgA responses to the gp41 HR1 region, a target of gp41/intestinal microbiota cross-reactive IgG antibodies described in normal and HIV-infected individuals [23, 48]. However, we could find no evidence that these cross-reactive antibodies were present in EC or other study subjects.

The possibility that humans have pre-existing polyreactive memory B cells that cross-react with gp41 was initially considered after it was discovered that the first HIV-specific IgA and IgG antibodies to appear in serum or secretions of HIV-infected individuals are directed against gp41 [59]. Subsequently, anti-gp41 IgG and IgA mAbs generated from circulating B cells or intestinal plasma cells of acutely-infected individuals were shown to react with lysates of aerobic and anaerobic intestinal bacteria [60], including a protein that was later identified as bacterial RNA polymerase [23]. Many of these antibodies were found to originate from class-switched IgM B cells and to have undergone somatic hypermutation, supporting the theory that they originate from memory B cells [23, 60]. Although the specificity of some gp41 cross-reactive antibodies is unknown, many have been mapped to the alpha subunit of *E*. *coli* RNA polymerase, which shares linear and conformational homologies with the gp41 HR1 region [48]. For this reason, we analyzed antibody responses to *E*. *coli* RNA polymerase and HR1 peptides containing the LRAI sequence. Neither IgG or IgA responses to RNA polymerase differed among our study subjects. The IgA responses to LRAI containing HR1 peptides also did not differ among EC, subjects on HAART and uninfected women. Thus, the higher magnitude IgA response and higher avidity IgA antibodies observed to the HR1 domain in EC do not exhibit characteristics of previously described gp41/microbiota cross-reactive antibodies.

Whether HR1-specific IgA antibodies could contribute in control of HIV infection is at this time unknown. Certainly, these antibodies do not neutralize, based on the absence of greater broadly neutralizing activity in serum of EC. However, the possibility that anti-HR1 antibodies could mediate phagocytosis by binding to gp41 stumps on HIV virions has not been tested. Antibody-mediated phagocytosis by non-neutralizing serum antibodies has been associated with delayed rates of infection acquisition and reduced viremia in nonhuman primate vaccine studies [61, 62]. EC also had strong IgA responses to the gp41 ID region. These antibodies could also potentially contribute in control of infection considering that anti-ID IgG mAbs can mediate phagocytosis of HIV particles by monocytes [14] and inhibit infection of macrophages [63, 64], presumably via phagocytosis. Anti-gp41 ID mAbs can also mediate ADCC [64, 65], and there is in vivo evidence that anti-ID antibodies could contribute in control of HIV infection. Passive administration of anti-ID human IgG mAbs to rhesus macaques has been found to reduce the number of founder viruses transmitted or acute viremia after rectal or vaginal challenge with SHIV [64, 66, 67]. As EC had stronger IgA responses to both the gp41 ID and HR1 regions when compared to HN, the possibility cannot be excluded that both ID- and HR1-specific IgA in EC might contribute to control of viral replication by mediating ADP or ADCC by monocytes, macrophages or neutrophils.

It is interesting that EC and subjects on HAART had highly discordant systemic IgG and IgA responses to gp70-V1V2. For EC and HAART recipients, 90% and 50% had IgG antibodies to Clade B gp70-V1V2, respectively. Infected subjects with strongest IgG responses to this scaffolded V1V2 protein demonstrated cross-clade reactivity to other gp70-V1V2 proteins with highly divergent sequences, a finding similar to that in the RV144 vaccine trial [40]. Thus, a large fraction of these antibodies may recognize conformational determinants or conserved sequences, such as the one in V2 that binds to α4β7 [68]. However, none of the HN and only 18–20% of EC and HC had IgA responses to the gp70-V1V2 Clade B protein. When they were detected, the levels of V1V2-specific IgA were very low, and no IgA responses to Clade A or C gp70-V1V2 proteins were found. Others have similarly found that only 17% of HIV-infected individuals have gp70-V1V2-specific IgA in serum or parotid saliva [69]. Whether these infrequent IgA responses to V1V2 are due to HIV infection is unclear. In the RV144 HIV vaccine trial, IgA responses to gp70-V1V2 were not reported for vaccine recipients, but the IgA response rates to V2 peptides were said to be too low to perform statistical comparisons [37]. Analysis of anti-gp70-V1V2 IgA antibodies induced by different vaccine modalities will be required to determine if IgA responses to V1V2 are also difficult to generate in healthy individuals.

We also observed discordant IgG and IgA antibody responses to C1 peptides with sequences proximal to the V1 loop. Both EC and HAART recipients rarely had anti-C1 IgG antibodies. However, many infected subjects had anti-C1 IgA. In addition, EC had stronger responses to C1 when compared to noncontrollers. This was an unexpected finding because high titers of C1-specific IgA in serum of RV144 vaccine recipients were associated with an increased risk of HIV infection [37]. It was also shown that IgA purified from plasma of RV144 vaccine recipients could interfere with ADCC mediated by IgG purified from these subjects [38]. Others have similarly found that removal of IgA from plasma of HIV-infected viremic individuals increased plasma ADCC activity against gp120-coated target cells [36]. However, ADCC activity in plasma of EC was not affected by IgA removal, even though the EC were found to have higher titers of anti-gp120 IgA binding antibodies [36]. Our results suggest that the lack of ADCC interference by EC IgA is unlikely to be due to lower concentrations of anti-C1 IgA or lower avidity anti-gp120 IgA antibodies in EC. However, other specificities of the anti-gp120 IgA and IgG antibodies in EC may differ from those in progressors. Additional studies aimed at determining why gp120-specific serum IgA antibodies in EC do not adversely effect IgG Fc-mediated antiviral functions may be warranted as they would benefit HIV vaccine development.

The increased frequency of IgA responses to T cell-dependent gag antigens in EC suggests that HIV-specific T helper cells which promote IgA responses may be better maintained in EC than in subjects who receive HAART during chronic infection. The types of TFH cells that promote class-switching to IgA have not been fully resolved in humans, but two subsets have been implicated in mice. These include TGF-β1-producing Foxp3^+^ T regulatory (Treg) cells that undergo conversion into Foxp3^-^ TGF-β1- and IL-21-producing TFH cells in lymphoid follicles in the gut [70]. However, this process may not be involved in the generation of systemic IgA responses as it has been reported to occur less efficiently in mesenteric and peripheral lymph nodes [70]. Other studies in mice have demonstrated that IL-21-producing Th17-type TFH cells can also promote mucosal IgA responses [71–74]. Importantly, adoptive transfer of Th17 cells to Th17-deficient mice increased serum IgA levels and numbers of IgA plasma cells in the bone marrow [71], suggesting that Th17 cells may also promote systemic IgA responses. Therefore, the loss of HIV-specific Th17 cells could potentially reduce an individual's ability to generate serum IgA antibodies to HIV. In this regard, both Th17 and Th17-type TFH cells have been reported to be preferential targets of HIV infection [75, 76]. Many HIV-infected individuals, including those on HAART [77], have reduced frequencies of Th17 cells in mucosal tissues [78, 79] and blood [80], and loss of these cells has been associated with disease progression [81]. However, EC have normal frequencies of Th17 in gut and blood [82–84]. Maintenance of IL-21-producing Th17 cells that induce class-switching to IgA might therefore explain the increased frequency of HIV-specific IgA responses in EC, as well as the lack of differences between EC and HC for HIV-specific IgG responses.

Despite the greater frequency of HIV-specific IgA responses in EC, we believe that production of antiviral IgA in these subjects may still be limited. This is suggested by the finding that the humoral response to HIV was dominated by IgG to a similar extent in EC and subjects on HAART. Studies with rhesus macaques suggest that it should be feasible to generate greater IgA responses to HIV. These animals similarly develop weak IgA antibody responses to SIV or SHIV after infection [53, 85]. However, if they are vaccinated before challenge, then macaques develop 100-fold greater SIV- or SHIV-specific IgA responses when infected [53, 86]. Intramuscular vaccination of humans or macaques with HIV Env proteins can also generate at least 10-fold greater Env-specific IgA responses than those observed in EC ([38] and Kozlowski, personal observations). Whether an IgA evasion mechanism such as inhibition of IgA class-switching by nef may be operative in EC is unclear. It has been reported that humans infected with nef-attenuated HIV viruses have higher levels of gp120- and p24-specific IgA in serum [28]. However, median levels of these antibodies were only roughly 3-fold greater than those in subjects infected with fully replication-competent HIV. Macaques infected with nef-deleted attenuated SIV also do not generate significantly greater SIV-specific IgA responses than animals infected with wild-type SIV [19]. Therefore, it seems more likely that predominant production of IgG antibodies in HIV infection might be related to unchecked production of cytokines or development of cells that promote class-switching to IgG, such as the Th1-biased TFH cells observed in SIV-infected macaques [87].

EC have been described as being immunologically heterogeneous because of their highly diverse gag-specific T cell responses, even among those with protective MHC alleles [3, 88]. As shown here, IgA antibody responses to HIV, like IgG [58], can also be quite variable in EC, especially those to p24 gag. Others have shown that IgG responses to HIV antigens do not differ between EC with or without protective B*57 or B*27 alleles, with the exception of higher anti-gp41 IgG2 in B*57+ subjects [58]. Instead, levels of total IgG1 best distinguished EC with high titers of HIV-specific IgG1 and polyfunctional anti-Env IgG antibodies from EC with lower titers and Fc-mediated functional activities, such as phagocytosis and ADCC. We similarly found that EC with elevated total IgG had stronger IgG responses to HIV. Expanding on previous observations, we found that the EC with IgG hypergammaglobulinemia also had stronger HIV-specific IgA responses. Whether this similarly translates into greater IgA functional activity is currently unknown. These differences in HIV-specific IgG or IgA responses between subjects with elevated versus normal total IgG were not observed in HN. Together, the results of these studies suggest that variable antibody responses in EC can be attributed to the development of a highly focused antiviral humoral response in some of these subjects, which results in IgG hypergammaglobulinemia. In contrast, elevated IgG in HN appears to reflect both specific and nonspecific B cell activation. As HN with elevated IgA typically exhibited the poorest HIV-specific IgG and IgA responses, IgA hypergammaglobulinemia may indicate that this balance has tipped towards nonspecific polyclonal B cell activation. This interpretation is consistent with previous studies showing that levels of total IgA are inversely associated with HIV-specific IgA responses in viremic subjects not on HAART [20] and other studies associating IgA hypergammaglobulinemia with immune activation [89, 90]. In this regard, it is intriguing that EC had lower concentrations of total serum IgA than Neg controls, and among EC, there was very little diversity in total IgA concentrations, as seen in another study with EC [8].

In conclusion, our data indicate that EC develop more frequent IgA responses to HIV gp160 and gag antigens, stronger IgA responses to the HR1 domain of gp41, and higher avidity gp41-specific IgA antibodies when compared to either viral suppressors or noncontrollers on HAART. Future functional analysis of the IgA antibodies produced by EC will be required to determine if they could contribute in control of HIV infection.

## Supporting information

S1 Table CD4 T cell counts and length of time on HAART. (PDF)

S2 Table Within group differences in IgA responses to HR1 peptides. (PDF)

S1 Fig Reactivity of IgG to HIVIIIB proteins. The percentage of infected subjects positive for IgG antibodies to each HIV protein was determined by WB using T1 and T2 serum samples. *p < 0.05 using the Fisher's exact test.
(TIFF)

S2 Fig Magnitude of the C1-specific IgA response using BAMA. The fluorescence intensity (FI) measured for IgA using 1/100 dilutions of IgG-depleted T2 serum samples and magnetic beads labeled with the C1 peptide MHEDIISLWDESLKPCVKLTPLCV was divided by the total IgA in the diluted serum. This value was then multiplied by the volume of serum tested (50 μl) to obtain the FI per μg total IgA. Results are presented as a Tukey box plot. *p < 0.05 by two-tailed Mann-Whitney rank sum test.
(TIF)

S3 Fig **Magnitude of IgG responses to gp70-V1V2 proteins with sequences representative of HIV Clades A, B and C**. The IgG sp. act. to gp70 scaffolded proteins with V1V2 sequences representative of **(A)** Clade B, **(B)** Clade C and **(C)** Clade A was measured using ELISA. Within each group, the same symbol is used to designate each subject. Dashed lines represent the cut-offs for significance determined using Neg controls.
(TIF)

S4 Fig Domination of the HIV-specific humoral response by IgG. For each infected subject, the **(A)** IgG or **(B)** IgA sp. act. measured against gp120, gp41BAL or p24 was divided by the corresponding mean sp. act. of Neg controls to determine the IgG and IgA responses in infected individuals. Black symbols and lines represent medians. The magnitude of antibody responses to each HIV antigen were compared by Mann-Whitney.*p < 0.05; **p < 0.01 and ***p < 0.001. **(C)** The magnitude of the IgG response to each antigen was divided by the magnitude of the corresponding IgA response. Bars denote medians. No significant differences were found between the groups for antigen-specific IgG/IgA ratios.
(TIF)

S5 Fig Correlation between HIV-specific IgA and IgG responses. The IgA and IgG sp. act. measured to gp120, gp41BAL, and p24 at T1 and T2 was compared for subjects within each infection group using logarithmically transformed values and the two-tailed Spearman Rank correlation test. Correlation coefficients and p values obtained are shown in each graph. In all cases, the IgA and IgG sp. act. were found to be significantly correlated.
(TIF)

S6 Fig Avidity of anti-HIV IgA and IgG antibodies at T1 and T2. The avidity indices for IgG (left panel) or IgA (right panel) antibodies to **(A)** gp120, **(B)** gp41BAL, **(C)** p24 and **(D)** gp70-V1V2 at T1 and T2 are depicted in minimum-to-maximum whisker box plots. The number of subjects with antibody concentrations high enough for avidity analysis at both time points is noted below each graph. Using the Wilcoxon matched pairs test, IgA and IgG avidity indices measured to each protein at T1 and T2 were not found to differ within any of the groups.
(TIF)

S7 Fig Correlation analysis of HIV-specific IgA and IgG antibody avidities. The avidity indices measured for gp120, gp41BAL and p24-specific IgA and IgG at T1 and T2 within each group were compared using the Spearman rank correlation test. Significant differences are indicated by p values and correlation coefficients in the graphs. ns: not significant.
(TIF)
