# Supplementary material for: Differences in serum IgA responses to HIV-1 gp41 in elite controllers compared to viral suppressors on highly active antiretroviral therapy
Source: PLoS One. 2017 Jul 3;12(7):e0180245. doi: 10.1371/journal.pone.0180245 (PMC5495342; doi:10.1371/journal.pone.0180245)
Supplement: S1 Table — (PDF) [file pone.0180245.s001.pdf]

**S1 Table. CD4 T cell counts and length of time on HAART**

| Subject | CD4 count before HAART (cells/μl)[a] | HAART duration (years)[b] | CD4 count at T1 (cells/μl) |
|---|---|---|---|
| HC1 | 280 | 3.8 | 849 |
| HC2 | 726 | 10.3 | 741 |
| HC3 | 288 | 9.4 | 1185 |
| HC4 | 336 | 1.0 | 424 |
| HC5 | 345 | 3.4 | 574 |
| HC6 | 211 | 3.8 | 577 |
| HC7 | 320 | 4.8 | 810 |
| HC8 | 288 | 7.6 | 590 |
| HC9 | 203 | 3.4 | 439 |
| HC10 | 207 | 8.0 | 412 |
| HC11 | 106 | 6.7 | 941 |
| HC12 | 84 | 7.3 | 451 |
| HC13 | 408 | 3.3 | 838 |
| HC14 | 545 | 5.4 | 495 |
| HC15 | 420 | 7.5 | 678 |
| HC16 | 619 | 4.8 | 673 |
| HC17 | 796 | 7.8 | 482 |
| HN1 | 345 | 6.4 | 55 |
| HN2 | 193 | 4.6 | 190 |
| HN3 | 207 | 7.6 | 29 |
| HN4 | 350 | 5.6 | 219 |
| HN5 | 95 | 3.1 | 79 |
| HN6 | 56 | 5.0 | 38 |
| HN7 | 231 | 3.6 | 28 |
| HN8 | 238 | 6.1 | 41 |
| HN9 | 215 | 3.5 | 82 |
| HN10 | 166 | 5.0 | 5 |
| HN11 | 299 | 2.9 | 109 |
| HN12 | 248 | 2.4 | 140 |
| HN13 | 264 | 6.7 | 102 |
| HN14 | 202 | 2.3 | 215 |
| HN15 | 172 | 4.0 | 282 |
| HN16 | 265 | 5.4 | 87 |
| HN17 | 218 | 4.9 | 282 |

[a] CD4 T cell count at the time when HAART was initiated.

[b] Years on at least 3 antiretroviral medications prior to T1.

The geometric mean length of time on HAART before T1 was 5.1 and 4.4 years for HC and HN, respectively.
