# Supplementary material for: Differences in serum IgA responses to HIV-1 gp41 in elite controllers compared to viral suppressors on highly active antiretroviral therapy
Source: PLoS One. 2017 Jul 3;12(7):e0180245. doi: 10.1371/journal.pone.0180245 (PMC5495342; doi:10.1371/journal.pone.0180245)
Supplement: S2 Table — (PDF) [file pone.0180245.s002.pdf]

S2 Table. Within group differences in IgA responses to HR1 peptides

Neg	P1	P2	P3	P4	P5	P6	P7	P8
P1	-	-	-	-	-	**	-	-
P2	-	-	-	-	-	*	-	-
P3	-	-	-	-	-	-	-	***
P4	-	-	-	-	-	-	*	***
P5	-	-	-	-	-	-	*	***
P6	**	*	-	-	-	-	***	***
P7	-	-	-	*	*	***	-	-
P8	-	-	***	***	***	***	-	-
EC								
P1	-	-	-	-	-	*	-	-
P2	-	-	-	-	-	-	-	-
P3	-	-	-	-	-	-	**	***
P4	-	-	-	-	-	-	*	**
P5	-	-	-	-	-	-	-	*
P6	*	-	-	-	-	-	***	***
P7	-	-	**	*	-	***	-	-
P8	-	-	***	**	*	***	-	-
HC								
P1	-	-	***	-	*	***	-	-
P2	-	-	-	-	-	*	-	*
P3	-	-	-	-	-	-	**	***
P4	-	-	-	-	-	-	-	**
P5	*	-	-	-	-	-	-	***
P6	***	*	-	-	-	-	***	***
P7	-	-	**	-	-	***	-	-
P8	-	*	***	**	***	***	-	-
HN								
P1	-	-	*	-	-	***	-	-
P2	-	-	-	-	-	-	-	-
P3	*	-	-	-	-	-	-	*
P4	-	-	-	-	-	-	-	-
P5	-	-	-	-	-	-	-	-
P6	***	-	-	-	-	-	***	***
P7	-	-	-	-	-	***	-	-
P8	-	-	-	-	-	***	-	-

A dash indicates no significant difference.

*p < 0.05, **p < 0.01 and ***p < 0.001 using Dunn's multiple comparison test.
