# Supplementary figures and images for: Differences in serum IgA responses to HIV-1 gp41 in elite controllers compared to viral suppressors on highly active antiretroviral therapy
Source: PLoS One. 2017 Jul 3;12(7):e0180245. doi: 10.1371/journal.pone.0180245 (PMC5495342; doi:10.1371/journal.pone.0180245)

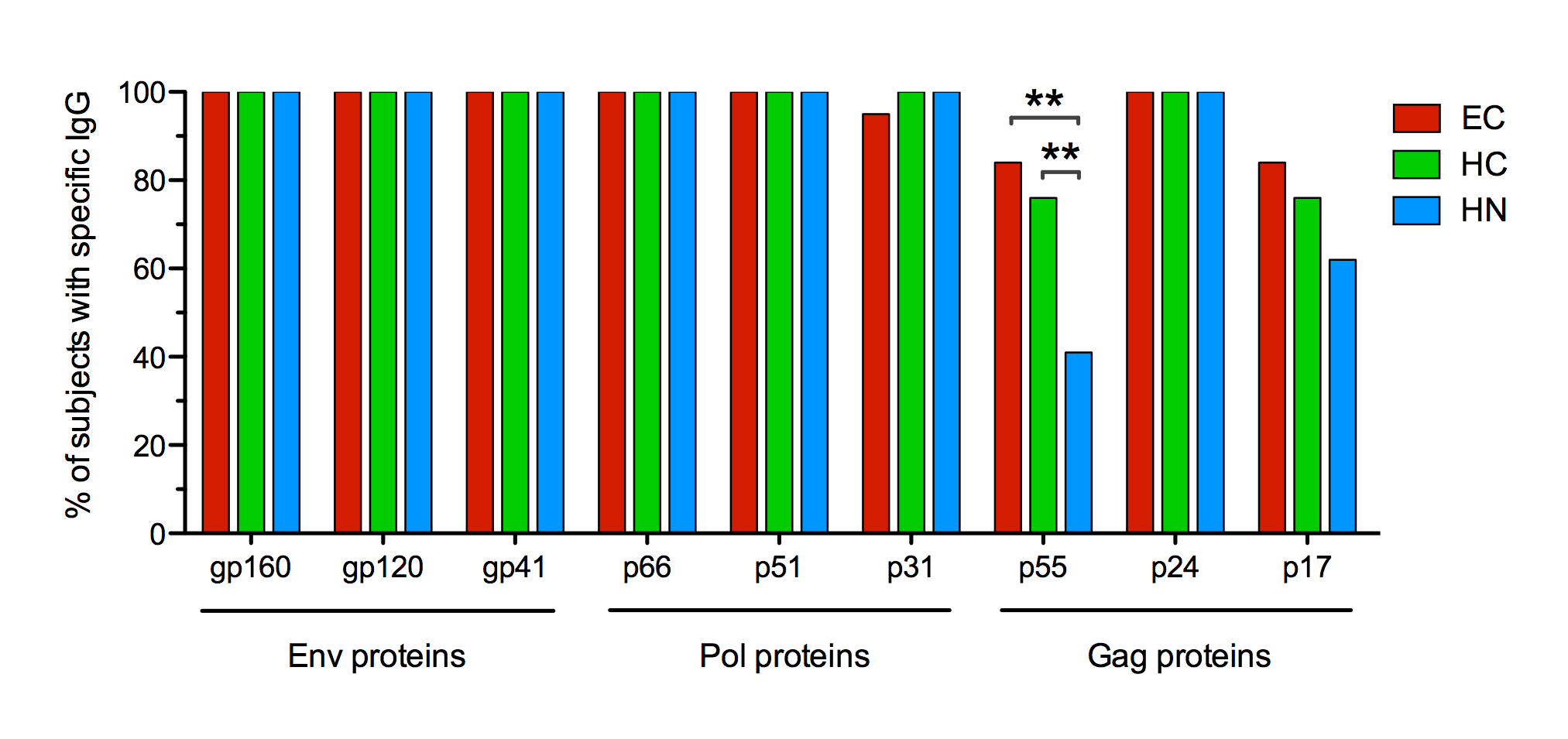

Supplement: S1 Fig — The percentage of infected subjects positive for IgG antibodies to each HIV protein was determined by WB using T1 and T2 serum samples. *p < 0.05 using the Fisher's exact test. (TIFF) [file pone.0180245.s003.tiff]

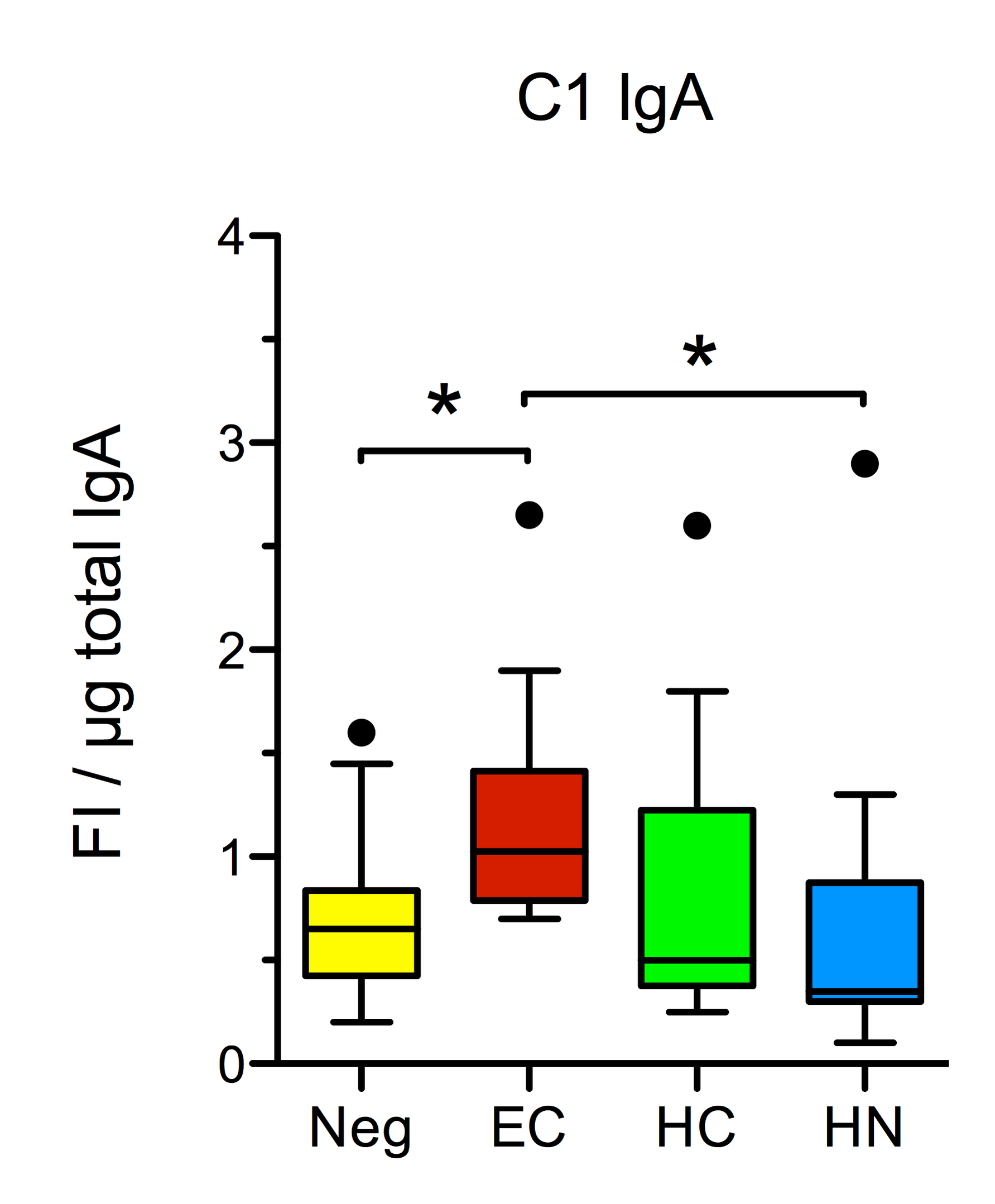

Supplement: S2 Fig — The fluorescence intensity (FI) measured for IgA using 1/100 dilutions of IgG-depleted T2 serum samples and magnetic beads labeled with the C1 peptide MHEDIISLWDESLKPCVKLTPLCV was divided by the total IgA in the diluted serum. This value was then multiplied by the volume of serum tested (50 μl) to obtain the FI per μg total IgA. Results are presented as a Tukey box plot. *p < 0.05 by two-tailed Mann-Whitney rank sum test. (TIF) [file pone.0180245.s004.tif]

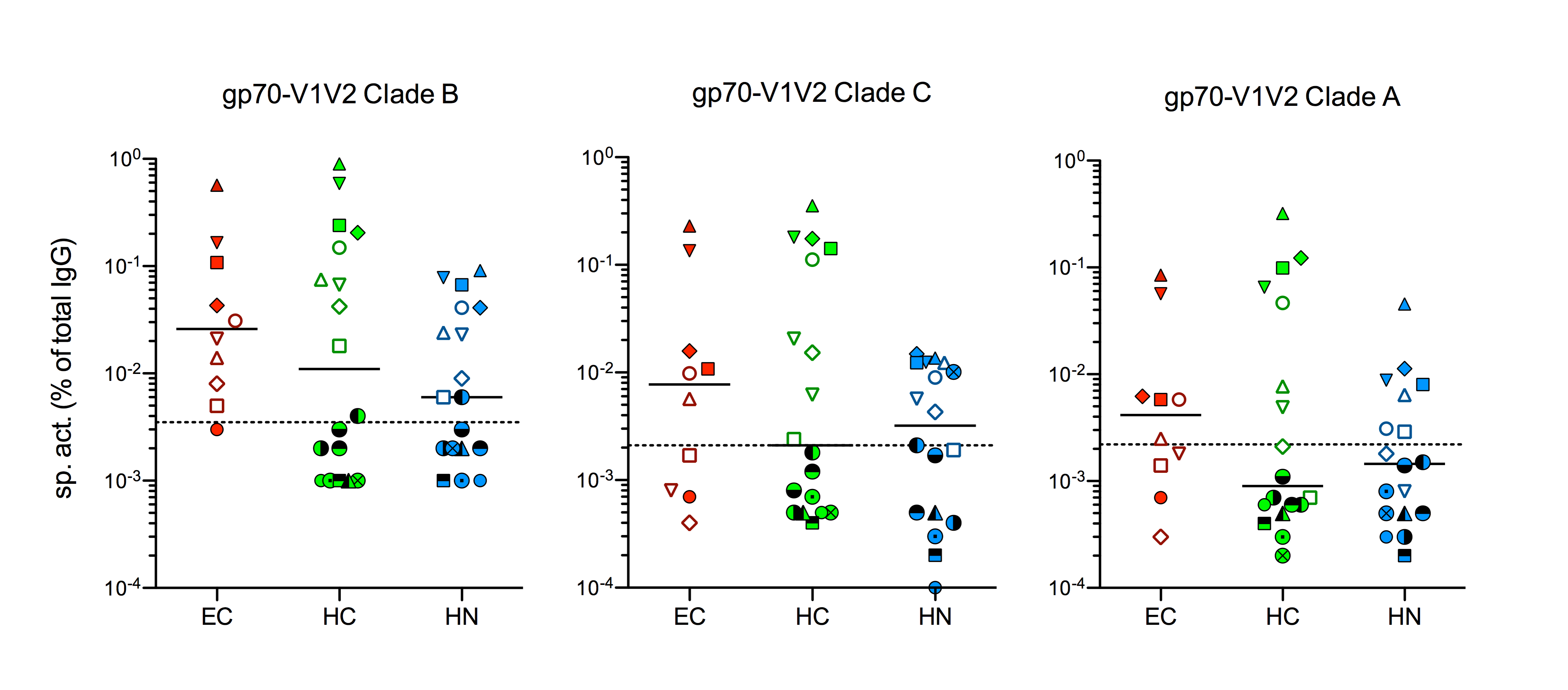

Supplement: S3 Fig — Magnitude of IgG responses to gp70-V1V2 proteins with sequences representative of HIV Clades A, B and C. The IgG sp. act. to gp70 scaffolded proteins with V1V2 sequences representative of (A) Clade B, (B) Clade C and (C) Clade A was measured using ELISA. Within each group, the same symbol is used to designate each subject. Dashed lines represent the cut-offs for significance determined using Neg controls. (TIF) [file pone.0180245.s005.tif]

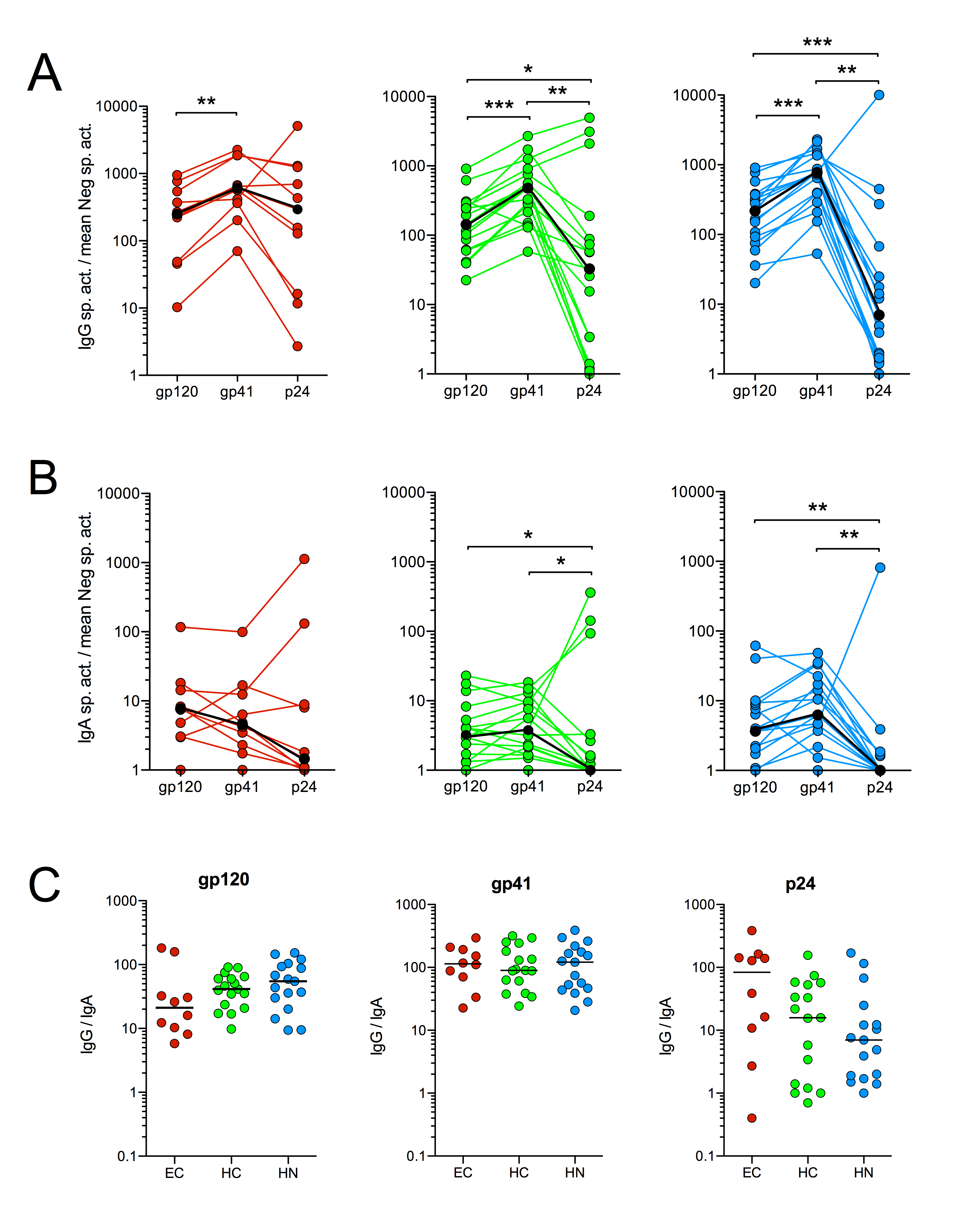

Supplement: S4 Fig — For each infected subject, the (A) IgG or (B) IgA sp. act. measured against gp120, gp41BAL or p24 was divided by the corresponding mean sp. act. of Neg controls to determine the IgG and IgA responses in infected individuals. Black symbols and lines represent medians. The magnitude of antibody responses to each HIV antigen were compared by Mann-Whitney.*p < 0.05; **p < 0.01 and ***p < 0.001. (C) The magnitude of the IgG response to each antigen was divided by the magnitude of the corresponding IgA response. Bars denote medians. No significant differences were found between the groups for antigen-specific IgG/IgA ratios. (TIF) [file pone.0180245.s006.tif]

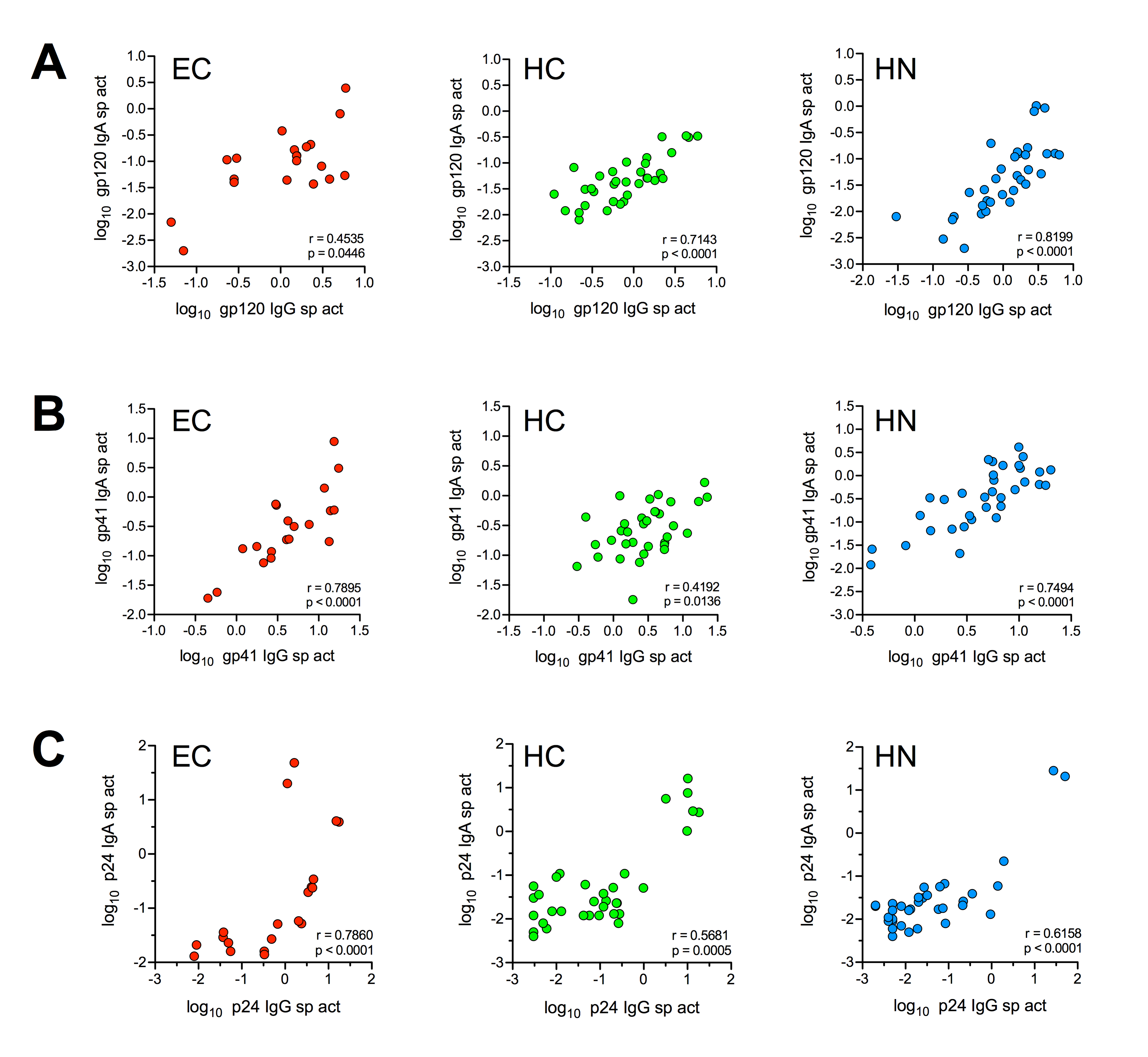

Supplement: S5 Fig — The IgA and IgG sp. act. measured to gp120, gp41BAL, and p24 at T1 and T2 was compared for subjects within each infection group using logarithmically transformed values and the two-tailed Spearman Rank correlation test. Correlation coefficients and p values obtained are shown in each graph. In all cases, the IgA and IgG sp. act. were found to be significantly correlated. (TIF) [file pone.0180245.s007.tif]

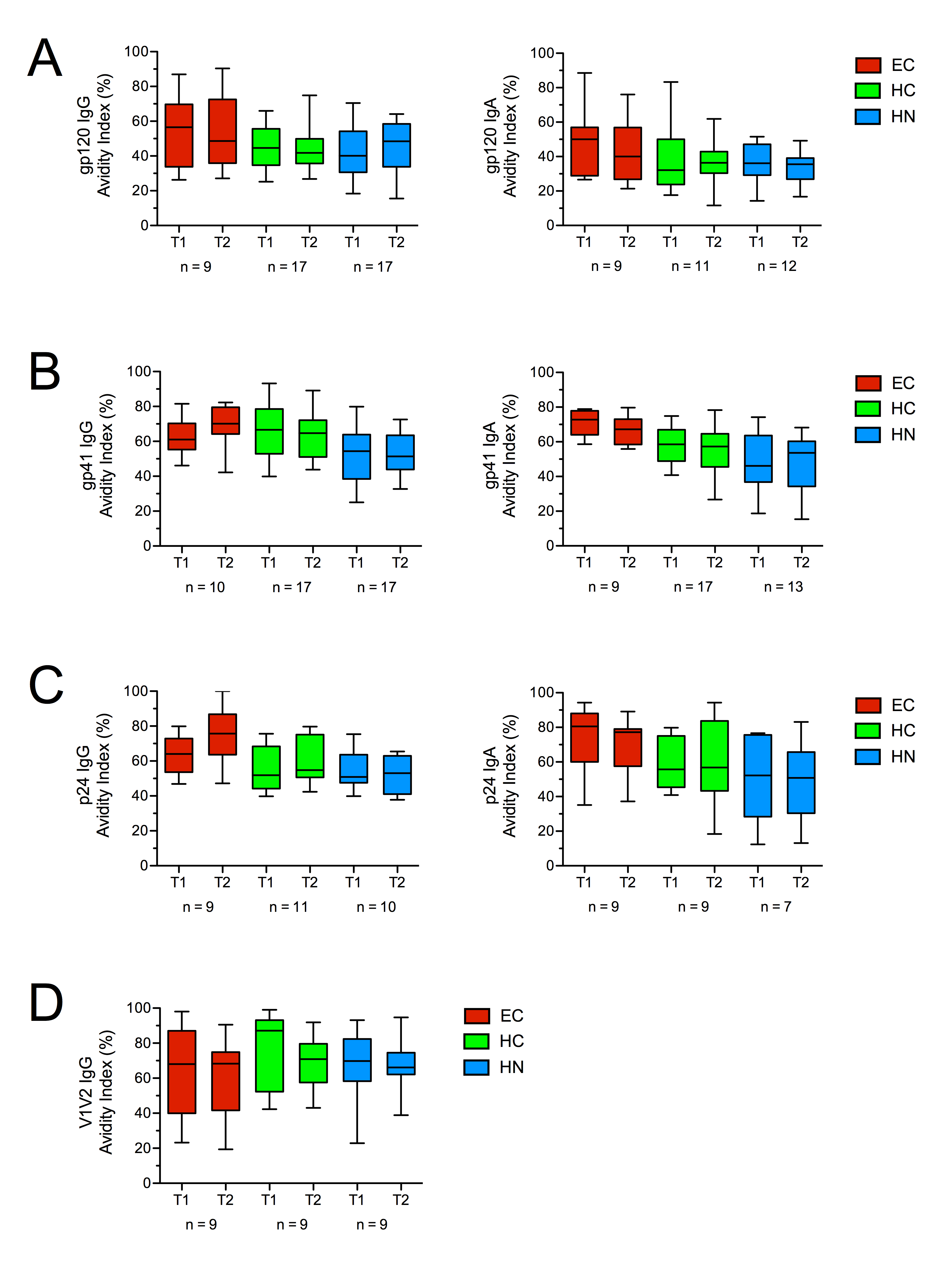

Supplement: S6 Fig — The avidity indices for IgG (left panel) or IgA (right panel) antibodies to (A) gp120, (B) gp41BAL, (C) p24 and (D) gp70-V1V2 at T1 and T2 are depicted in minimum-to-maximum whisker box plots. The number of subjects with antibody concentrations high enough for avidity analysis at both time points is noted below each graph. Using the Wilcoxon matched pairs test, IgA and IgG avidity indices measured to each protein at T1 and T2 were not found to differ within any of the groups. (TIF) [file pone.0180245.s008.tif]

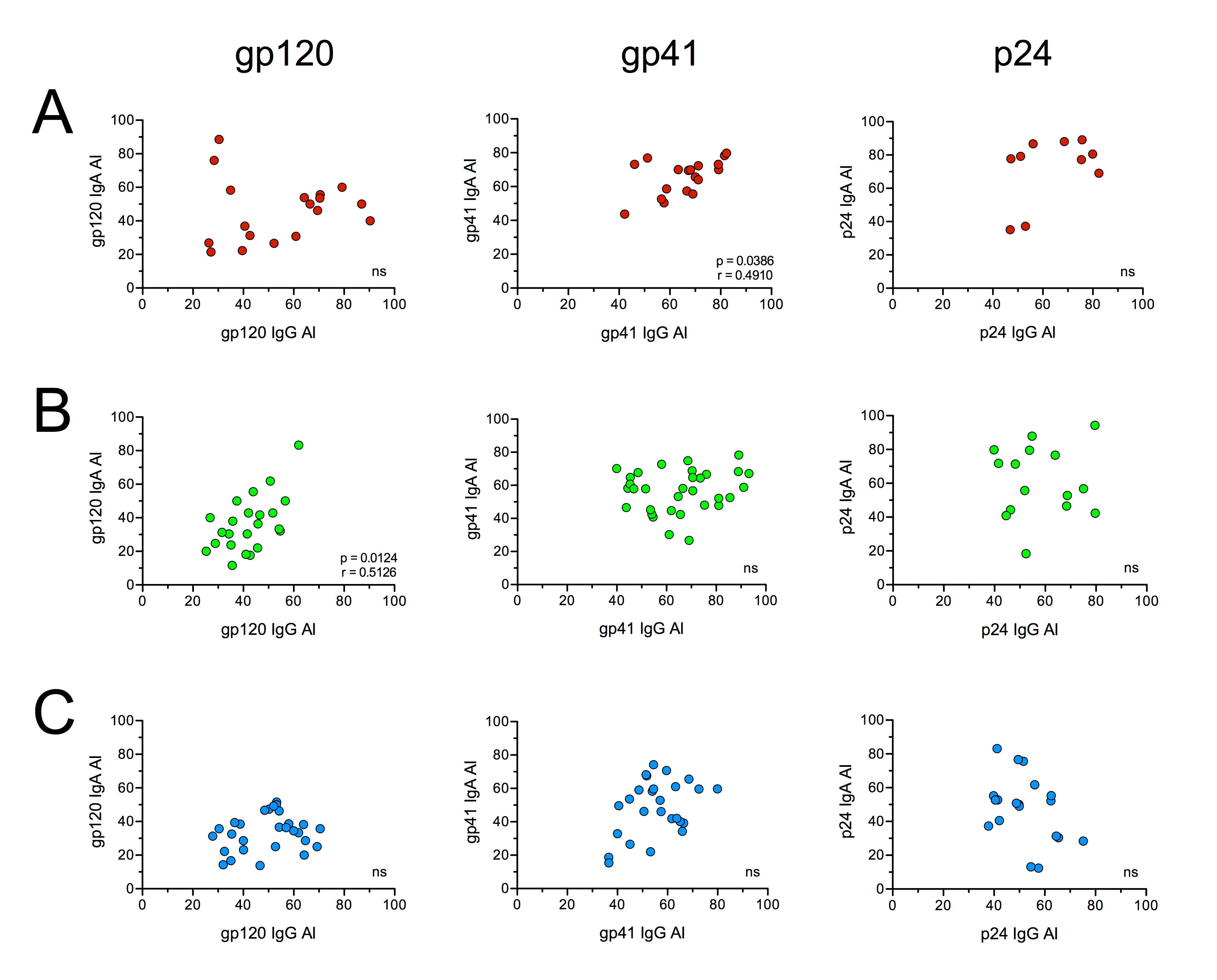

Supplement: S7 Fig — The avidity indices measured for gp120, gp41BAL and p24-specific IgA and IgG at T1 and T2 within each group were compared using the Spearman rank correlation test. Significant differences are indicated by p values and correlation coefficients in the graphs. ns: not significant. (TIF) [file pone.0180245.s009.tif]
